# The Prevalence of *H. pylori* Among Jordanian Type 2 Diabetic Patients and Its Association with ABO Blood Group

**DOI:** 10.3390/medicina61122167

**Published:** 2025-12-05

**Authors:** Hafez Al-Momani, Amro Bani-Hani, Ahmad A. Jaber, Azhar Alsmady, Yusra Sobh, Bassam Otoom, Iman Aolymat, Ashraf I. Khasawneh, Hala Tabl, Ayman Alsheikh, AbdelRahman M. Zueter, Abdel-Ellah Al-Shudifat

**Affiliations:** 1 Department of Microbiology, Pathology and Forensic Medicine, Faculty of Medicine, The Hashemite University, Zarqa 13133, Jordan; ashrafkh@hu.edu.jo (A.I.K.); halaa_mo@hu.edu.jo (H.T.); 2 Faculty of Medicine, The Hashemite University, Zarqa 13133, Jordan; dr.amrofbh@gmail.com (A.B.-H.); amdjbr99@gmail.com (A.A.J.); azharalsmady@gmail.com (A.A.); sobuhu@gmail.com (Y.S.); bassams.otoom123@gmail.com (B.O.); 3 Department of Anatomy, Physiology and Biochemistry, Faculty of Medicine, The Hashemite University, Zarqa 13133, Jordan; imank@hu.edu.jo; 4 Department of Medical Laboratory Sciences, Faculty of Allied Medical Sciences, Zarqa University, Zarqa 13132, Jordan; asheikh@zu.edu.jo; 5 Department of Medical Laboratory Sciences, Faculty of Applied Medical Sciences, The Hashemite University, Zarqa 13133, Jordan; zeuterabdelrahman@gmail.com; 6 Department of Internal Medicine, Neurology, Psychiatry and Dermatology, Faculty of Medicine, The Hashemite University, Zarqa 13133, Jordan; abdel-ellah@hu.edu.jo

**Keywords:** *Helicobacter pylori*, type II diabetes mellitus, ABO blood group, insulin resistance, Jordanian population, epidemiology

## Abstract

*Background and Objectives*: There is no universal agreement with regard to the correlation between *Helicobacter pylori* (*H. pylori*) infection, type 2 diabetes mellitus (T2DM), and ABO blood group antigens. The data related to these are limited. The purpose of this study is to explore the correlation and frequency of *H. pylori* infection with T2DM and ABO blood group of adults that reside in Jordan. *Materials and Methods*: This study adopts a cross-sectional comparison of 149 patients diagnosed with T2DM and 168 non-diabetic controls. The One-Step Immunochromatographic DiaSpot^®^ test was used to diagnose *H. pylori*, while standardized hemagglutination through the use of monoclonal anti-A, anti-B, and anti-D reagents was used for ABO blood grouping. Analyses were conducted on the correlation between *H. pylori* infection, diabetes, and ABO blood group through logistic regression. *Results*: A total of 89 out of the 317 participants tested positive for *H. pylori* infection (overall seroprevalence = 28.0%), consisting of 51 of the 149 T2DM patients (34.2%) and 38 (22.6%) of the 168 non-diabetic controls. A significant association was observed between diabetes status and *H. pylori* infection (χ^2^(1) = 4.71, *p* < 0.05), with the probability of being *H. pylori*-positive 1.78 times higher among diabetics (95% CI: 1.085–2.921). A significant association was found between blood group and *H. pylori* infection, (χ^2^(3), *n* = 317) = 15.01, *p* < 0.001. Of the 89 *H. pylori*-positive patients, 21 (23.6%) were in blood group A, 13 (14.6%) in group B, and 44 (49.4%) in group O, with the remaining 11 (12.4%) patients in blood group AB. *Conclusions*: Significant associations were found between *H. pylori* infection and both T2DM and blood type. Further longitudinal studies that include larger, more diverse populations and more potentially significant factors are needed to clarify these relationships.

## 1. Introduction

*Helicobacter pylori* infection is one of the most common global bacterial infections, with an estimated over 50% of the population infected, especially in developing regions [1]. There is a reported 50% to 70% of the population within the Middle East and North Africa (MENA) infected, influenced by environment, demographic, and socioeconomic factors [2,3,4,5]. There is a view that *H. pylori* infection increases the risk of cardiovascular conditions, insulin resistance, and metabolic syndrome, with possible causal pathways that involve elevations in inflammatory markers such as C-reactive protein (CRP) and Interlukin-6 (IL-6) [6,7,8]. Furthermore, some research implicates *H. pylori* in metabolic disease through its effects on the hormonal control of metabolic homeostasis. *H. pylori*, for example, is thought to affect the regulation of the two gastric hormones leptin and ghrelin, which play a vital role in insulin sensitivity and glucose homeostasis [9,10]. In diabetic patients, hyperglycemia can result in major organ damage and low level of immunity, which significantly increases the risk of infection by pathogenic bacteria [10]. There is evidence that type 2 diabetes mellitus (T2DM) patients have a higher risk of *H. pylori* infections as a result of a weakened immune system and increased systematic inflammation; however, it must be highlighted that inconsistencies between *H. pylori* infection and T2DM remain across different studies [11,12].

Despite increased research interest in *H. pylori*’s impact on metabolism, particularly on diabetes-related disease, there is currently no clear evidence indicating that this bacterium plays a causative role in such diseases. In fact, whether the prevalence of *H. pylori* infection is higher in diabetic patients remains controversial, with some studies [13,14] linking higher prevalence to diabetes, while some do not draw the same conclusion [15,16].

Such conflicting evidence is also found in research on blood group and *H. pylori* infection. Blood group is an important factor in susceptibility to infection because antigens specific to different blood groups act as receptors for lectins present on the exterior cell surfaces of many pathogenic microorganisms. The binding of microbial toxins facilitate invasion and subsequent colonization [17,18]. While Khosravi and Sirous [19] found individuals with O-type blood were most susceptible to *H. pylori* infection and those with type AB the least, their results conflict with data presented by Mahaseth and Mishra [20], which indicated that individuals with type B blood were the most susceptible. If a current consensus exists, it would be that individuals with blood groups O and B have a higher risk of *H. pylori* infection, while those with type AB are at a lower risk, suggesting that the latter blood type may have a protective effect.

Evidently, the relationships between *H. pylori* infection, diabetes, and blood group are still poorly understood, with many plausible mechanisms suggested as a means for these bacteria to exert an influence on disease, but with limited solid evidence pertaining to its role. Understanding these complex relationships in greater detail requires significant well-planned research, which could also provide insights into metabolic disease pathogenesis and therapeutic approaches more precisely tailored to individual genotypes.

In Jordan, T2DM is a main public health challenge and the cause of high healthcare costs as a result of complications. It is clinically relevant and important to comprehend possible coexisting infections such as *H. pylori*. Additionally, identification of host-related susceptibility factors, including the ABO blood group, may contribute towards specific screening or preventative strategies in Jordan’s healthcare system.

Jordan is a significant medical context in the study of *H. pylori* in terms of metabolic disorders due to the high prevalence of *H. pylori* infection and diabetes mellitus (DM). Estimates by the national health report suggests over 20% of adults in Jordan are diagnosed with DM, and rates forecasted to increase due to changes in lifestyle and demographics. Simultaneously, there is an increase in T2DM in Jordan with over 20% of adults diagnosed with it, which results in significant pressure upon the national healthcare system [21].

Furthermore, previous studies focusing on Jordan have forecasted the estimated value of *H pylori* infection to be 40% for asymptomatic cases and up to 70% for dyspeptic or high-risk clinical groups [4,22]. Simultaneously, studies in nearby regions suggest that the prevalence of *H. pylori* is persistent within the moderate to high range in Middle East populations, estimated to be from 50% to 70% depending on the socioeconomic status and diagnosis methods.

Despite several studies investigating the prevalence of *H. pylori* in general or dyspeptic populations in neighboring Middle Eastern and Mediterranean countries, including Turkey, Yemen, Saudi Arabia, and Egypt, few studies completely focus on patients diagnosed with T2DM, and even fewer studies specifically focus on the simultaneous role of the ABO blood group [23,24,25]. Additionally, the adoption of present diagnostic methods using latest, population-specific data obtained from Jordan remains limited. Therefore, this study widens the regional understanding through estimating the local prevalence of *H. pylori* in Jordanian patients diagnosed with T2DM. This study also investigates the association with ABO blood type within the aforementioned clinical subgroup. The identification of potentially indicative correlations would be valuable for both targeted interventions and screening procedures for individuals at high risk of developing diabetes.

## 2. Methods

### 2.1. Study Design

This research was a cross-sectional comparative study involving 149 patients diagnosed with T2DM and 168 healthy individuals without diabetes (control). All participants were over the age of 18, and the study was conducted at a Jordanian tertiary care hospital from April 2024 to August 2024.

T2DM status was accorded following the criteria given by the American Diabetes Association, which requires one or more of the following: (i) fasting blood sugar (FPG) ≥ 7.0 mmol/L; (ii) glycated hemoglobin A1c (HbA1c) ≥ 6.5%; and (iii) self-reported diagnosis of diabetes by a physician or the use of anti-diabetic medication. Participants were excluded if they fulfilled any of the following criteria: a diagnosis of type I diabetes; use of antibiotics, proton pump inhibitors, H2 receptor blockers, or antacids in the past four weeks; or any current or previous evidence of gastrointestinal bleeding, jaundice, or post-gastric surgery.

Controls were verified to be free of T2DM by random blood sugar and HBA1C screening. The other exclusion criteria applied for the controls were the same as those for the T2DM group. *H. pylori* infection status for both groups was determined through serology.

### 2.2. Sample Size

To calculate the sample size, historical serology-based *H. pylori* prevalence estimates (80–90%) that were available during the planning stage were employed. This was important because there is currently a lack of contemporary stool antigen-based data in Jordan. Thus, the calculation reflects an approximate planning estimate as opposed to a strict hypothesis-driven requirement [22]. The expression *n* = Z^2^P(1 − P)/d^2^ was used to determine *n*, where Z is the Z statistic for a given confidence interval, P is the assumed prevalence, and d is the margin of error or precision. For a 95% confidence level (Z = 1.96), a margin of error or absolute precision of ±5% (d = 0.05), and an assumed prevalence of 88% (P = 0.88), we calculated the minimum sample size to be 162 participants. However, considering potential missing values, invalid responses, or other reasons for data loss, we used a sample size about 10–20% larger than the calculated value of *n*, which is around 180–195 participants.

The assumption of an 88% prevalence rate was in accordance with prior reports that were drawn from populations in the Middle East through serological testing, where previous data showed prevalent seropositivity among adults diagnosed with chronic conditions [26,27]. The reason for the adoption of an upper-bound estimate was to ensure sufficient statistical power for subgroup analysis to be conducted on the ABO blood group. However, due to dynamic changes in population and diagnostic methods, recent studies have shown decreased prevalence levels, which is in accordance with the 28% prevalence in this study, based on stool antigen-based rapid immunoassay.

Despite a higher prevalence being indicated by the original estimate, the final sample size was calculated as *n* = 317. This means there is 90% power to identify observed differences in prevalence between diabetics and controls (28% vs. 18%) at α = 0.05, and 80% power when using multivariable modeling with the included predictors (rule-of-thumb ≥10 events per parameter). The findings of the post hoc evaluation revealed that the sample size was adequate for supporting the primary analyses and multivariable regression models according to the observed event rate.

### 2.3. Sample Characteristics

Participants who had given their informed consent were asked to complete an investigator-designed structured questionnaire that was exclusive for this study. The questionnaire was completed face to face by trained medical researchers, who were supervised by a principal investigator to maintain accuracy and consistent data collection. The questionnaire obtained demographic and lifestyle data such as age, gender, height, weight, smoking status (smoker/non-smoker), and highest academic achievements (illiterate, primary, secondary, bachelor’s degree, or postgraduate). Reliability was maintained through cross-checking the responses with participants’ medical and laboratory whenever possible. The completeness of the data was observed constantly, including during data entry. Body mass index (BMI) was calculated by dividing weight (kg) by height squared (kg/m^2^).

### 2.4. Blood Sample Collection and Processing

Participants gave their blood samples (venous, min 5 mL) using vacutainers, which were administered by a nurse. To confirm that control participants were not diabetic, an HbA1c test was performed by adding approximately 2.5 mL of their blood to ethylene diamine tetra acetic acid (EDTA) in vacutainer tubes. Each control’s blood HbA1c level was determined through ion-exchange chromatography using high-performance liquid chromatography (HPLC) on a Bio-Rad D-10 Hemoglobin Testing System (Bio-Rad Laboratories, Hercules, CA, USA), and the percentage of glycated hemoglobin was assessed with spectrophotometer at 450 nm. The test sensitivity (detection limit) was 0.0151%. T2DM status was assigned if the HbA1c level was higher than the 6.5% cutoff value as recommended by the American Diabetes Association [28].

In order to test for *H. pylori* infection, serum samples were obtained by placing 2.5 mL of sample in tubes without anticoagulant. The samples were left for 10 min at room temperature to allow for clotting and were then centrifuged at 3000 rpm for 10 min. Three drops of serum were then analyzed through rapid chromatographic immunoassay using a DiaSpot^®^ *H. pylori* One Step Test Device (PT Indo DiaSpot, Jakarta, Indonesia) (Supplementary Figure S1). Manufacturer instructions were strictly adhered. This test provides a rapid (~10 min) result with sensitivity of more than 95.9%, specificity ~75.9%, and an accuracy of 85.2%, in comparison with histological *H. pylori* samples obtained by endoscopy. The test was performed by strictly adhering to the instructions that were provided. The test produces a colored line on the test strip, with one line indicating negative (*H. pylori* not detected) and two lines indicating positive (*H. pylori* detected).

Since the rapid immunochromatographic test detects anti-*H. pylori* IgG, there is a possibility that it may not distinguish between active and prior infection as IgG may remain after eradication or previous exposure [29,30]. Furthermore, the moderate specificity (~75.9%) of the assay presents a possibility of false-positive classification [29]. These diagnostic attributes were taken into consideration during the determination of estimates and associations.

### 2.5. Expression of ABO Antigens in Blood

Standardized hemagglutination tests were used to determine ABO blood group antigens using anti-A, anti-B, anti-AB, and anti-D monoclonal antibodies, while adhering to the manufacturer’s recommended procedure (Bio-Rad, Hercules, CA, USA).

### 2.6. Ethics

All procedures adhered to the standards of the 1975 Declaration of Helsinki. Ethical approval was granted by the Hashemite University Ethical Committee (Approval no. 1/4/2022/2023). Potential participants were provided with written and oral information detailing the purpose and procedures that were involved in the study, how their blood samples would be used, the potential risks and benefits of the research, the voluntary nature of participation, their right to withdraw at any time, and the measures taken to ensure confidentiality. Participants were only enrolled after they gave their voluntary signed consent.

### 2.7. Statistical Analyses

SPSS (version.20) (IBM Corp., Chicago, IL, USA) was adopted to conduct all statistical analyses. Unpaired *t*-tests were conducted to compare continuous variables between groups. Where suitable, Chi-square or Fisher’s exact tests were conducted for categorical variables. Odds ratios (ORs) with 95% confidence intervals were calculated to assess associations between *H. pylori* infection, T2DM, and demographic variables. A subset of participants was used as a reference group for comparison with other groups and was defined as those aged ≥60 years, having a BMI ≥ 25 kg/m^2^, and testing positive for *H. pylori*. Relationships involving *H. pylori* infection and T2DM with *p*-values below or close to 0.05 were further evaluated through logistic regression. Multivariate logistic regression models were formed to control for potential confounding by adjusting for age, gender, BMI, smoking status, and education level.

Bonferroni correction was conducted for the purpose of adjusting the significance threshold in order to decrease the risk of Type I error arising from multiple subgroup comparisons, such as age, education level, BMI, and smoking status. The expression for alpha was α = 0.05/number of comparisons, with adjustment threshold presented in the Section 3. All analyses were conducted with a significance value of *p* ≤ 0.05.

## 3. Results

### 3.1. Participant Characteristics

There was a total of 317 participants that took part in the study, of whom 149 had T2DM and the remaining 168 were non-diabetic controls. Demographic details of the participants are shown in Table 1. The mean age of diabetic participants (57.8 ± 12.8 years) was significantly higher than that of controls (43.1 ± 13.4 years). The diabetic and control groups also had significantly different distributions of age groups (χ^2^ = 78.73, *p* ≤ 0.05), with the diabetic group possessing a higher proportion of older participants.

The diabetic group had a higher proportion of male participants, with 61.7% more than the control group (51.8%), but the difference was insignificant (χ^2^ = 2.79, *p* = 0.09). The proportion of smokers in the diabetic group (47.0%) was higher than in the control group (35.7%), and this difference was marginally significant (χ^2^ = 4.19, *p* ≤ 0.05, OR = 1.60, 95% CI: 1.02–2.50). Diabetics had a significantly higher average BMI (30.77 ± 5.85) than non-diabetics (28.45 ± 5.36), (t = 3.19, *p* ≤ 0.05). Although T2DM rates were similar across different education levels, they were significantly higher among those with a lower education level (such as illiterate, primary, and secondary), among whom 67.8% had T2DM (*p* ≤ 0.05).

A shown in Table 2, the significant difference in age distribution reflected the higher relative proportions of individuals aged 60–69 years and 70–79 years within the diabetic group (OR = 2.80, 95% CI: 1.69–4.66 and OR = 9.25, 95% CI: 2.12–40.35, respectively) in addition to a lower relative proportion in the youngest age group (20–29 years) (OR = 0.096, 95% CI: 0.03–0.32).

Diabetics were also relatively over-represented in the higher BMI categories (BMI ≥ 30). The odds ratios for being diabetic increased in order from the lower BMI categories (ORs < 1) to the higher (OR of 1.73 for BMIs between 35 and 29.9). Despite the clear trend, these differences were not significant (all *p*-values ≤ 0.05).

The diabetic group had disproportionately more smokers than the control group (35.7% vs. 47.0%; χ^2^ = 4.19, *p* ≤ 0.05). Thus, it is assumed that smokers have a higher risk of diabetes (OR = 1.60, 95% CI: 1.02–2.50).

There was a strong relationship between level of education and diabetes status. The proportion of diabetics who were illiterate (7.4%) or had only received primary education (20.1%) was higher than the controls (1.2% and 95%, respectively), with illiterate participants having a higher risk of diabetes (OR = 6.62, 95% CI: 2.71–49.89).

### 3.2. Factors Associated with Diabetes: Logistic Regression Analyses

Logistic regressions were performed in this work to further examine the relationship between lifestyle/demographic factors and diabetes. The findings revealed that the variables of high BMI, smoking, low educational background, and older age in the multivariable logistic regression model were independently related to T2DM. Participants aged 40–49 (aOR = 0.39, 95% CI: 0.23–0.66, *p* ≤ 0.05), 30–39 (aOR = 0.29, 95% CI: 0.13–0.60, *p* ≤ 0.05), and 20–29 years (aOR = 0.11, 95% CI: 0.03–0.36, *p* ≤ 0.05) had significantly lower chances of developing diabetes than those aged 60 and over. Moreover, obesity was found to be independently associated with diabetes, with the highest adjusted odds being reported for those with a BMI ≥ 35 kg/m^2^ (aOR = 2.42, 95% CI: 1.21–4.86, *p* ≤ 0.05). Current smoking status was also significant (aOR = 1.51, 95% CI: 1.03–2.23, *p* ≤ 0.05). Illiterate individuals or those who had only participated in primary education had more than double the odds of developing diabetes than individuals who had achieved secondary education certificates (aOR = 2.28, 95% CI: 1.28–4.02, *p* ≤ 0.05). After adjustments were made, gender was not found to be statistically significant (*p* = 0.11) (Table 3).

There was statistically significant evidence (*p* < adjusted α = 0.05) when Bonferroni correction was applied to include the multiple subgroups comparisons such as associations between age group, level of education, and smoking status with T2DM. Older participants aged ≥ 60 years and those with lower levels of education consistently demonstrated strong positive associations with diabetes. Participants who smoked had a modest yet significant effect. In comparison, despite a similar trend in the associations between BMI and T2DM, they remained insignificant post-correction (adjusted *p* > 0.0125). The aforementioned adjusted findings are presented in Table 4.

### 3.3. H. pylori Infection Rates in T2DM Participants and Non-Diabetics

**Figure 1:**
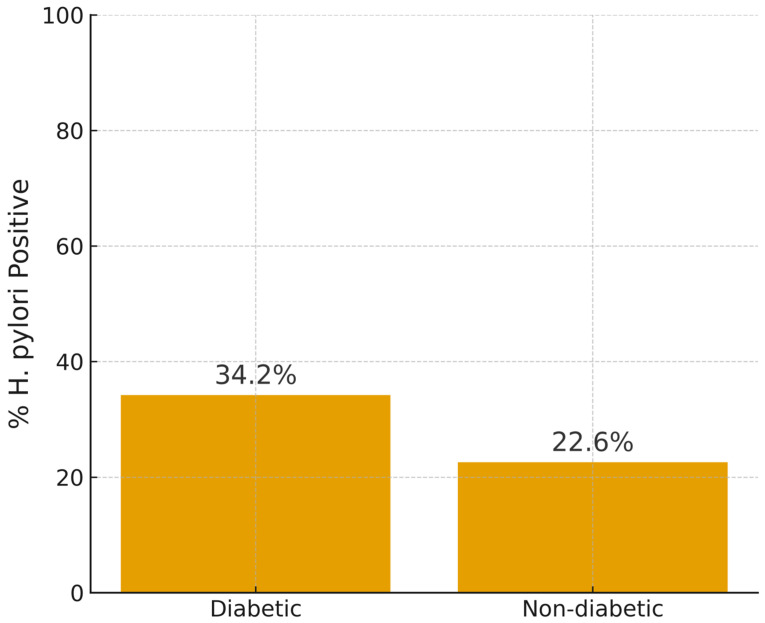
*H pylori* positivity status rate according to T2DM status.

The 89 participants who tested positive for *H. pylori* infection represented 28% of the total participants (317), of which 51 were diagnosed with T2DM and 38 were non-diabetic. In terms of rates within the diabetes status categories, these positive cases represent 34.2% of the T2DM group and 22.6% of the non-diabetics (Figure 1). The association between *H. pylori* infection and diabetes status was significant but not strong (χ^2^ = 8.30, df = 1, *p* ≤ 0.05, Cramér’s V = 0.122), with diabetics being 1.78 times more likely to be infected than non-diabetics (95% CI: 1.08–2.92) (Table 5).

An examination of the associations between the lifestyle/demographic variables and *H. pylori* infection status using Chi-square tests demonstrated that both smoking status and education level had significant effects. Being a smoker increased the odds of *H. pylori* infection from 0.70 in non-smokers (95% CI: 0.48–1.00) to 1.55 (95% CI: 1.07–2.25), (χ^2^ = 8.30, df = 1, *p* ≤ 0.05). Furthermore, there was an association between *H. pylori* infection rate and education level (χ^2^ = 7.30, df = 3, *p* ≤ 0.05), with those in the lower education groups more likely to be infected than those with more education. An individual educated up to primary level had a probability of being infected at 2.20, 95% (CI: 1.22–3.96) compared with 0.68 (95% CI: 0.43–1.09) for an individual that held a bachelor’s degree (although not significant). The increased risk of infection among the poorly educated individuals reflects multiple factors correlated with education level, most of which can be attributed to income/socioeconomic status.

No observations were made on associations between age (χ^2^ = 10.35, *p* = 0.111), gender (χ^2^ = 0.16, *p* = 0.66), or BMI (χ^2^ = 10.54, *p* = 0.06) and *H. pylori* infection within the study sample. The odds ratios infections for males and females were 0.93 (95% CI: 0.66–1.32) and 1.09 (95% CI: 0.75–1.59), respectively. There was a trend that higher BMI resulted in greater risk of infection; however, the association was weak.

### 3.4. H. pylori Infection Rates in ABO Blood Groups

**Figure 2:**
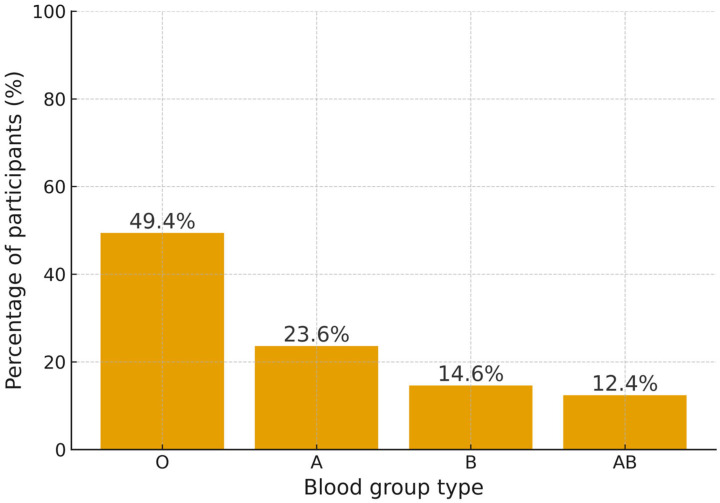
*H. pylori* positivity rates according to ABO blood groups. Axes have been relabeled with clear titles (X-axis: percentage of participants (%) and Y axis: blood group type).

Of all the 89 participants who were tested positive for *H. pylori*, 44 (49.4%) had blood group O, 21 (23.6%) A, 13 (14.6%) B, and 11 (12.4%) AB. Figure 2 shows the *H. pylori* infection rates within each ABO blood group. A Chi-square test indicated a weak-to-moderate dependence of *H. pylori* infection on blood group (χ^2^(3), *n* = 317) = 15.01, *p* ≤ 0.05, Cramer’s V = 0.218) (Table 6).

## 4. Discussion

The question of whether there is a connection between T2DM and *H. pylori* infection remains open and has been debated for many years. Some studies, such as Yu and Wei [31] and Wawro and Amann [32], found no evidence of an association, while others [33,34,35] have observed positive associations. One study related to this question [13], which used a meta-analysis of 41 case-controlled studies, had no substantial conclusion that infection with *H. pylori* may increase the risk of T2DM. The assessed studies included two conducted in China, where no association was found.

The overall picture is further complicated by Chen and Xing [36], where meta-analysis confirmed the plausibility of an *H. pylori*–diabetes link. Evidence has revealed that this bacterium can increase diabetic patients’ levels of HBa1c, therefore contributing towards metabolic dysregulation characteristic of diabetes. The extent of *H. pylori’s* involvement in diabetic individuals is unlikely to be fully understood for some time. Studies (e.g., [33,34,35,37]) continue to implicate it as a risk factor while also advocating caution about oversimplifying the putative relationship. The evidence thus needs to be taken on its own terms, with both an association and a lack of one entirely plausible given the host of additional factors—particularly demographic ones—involved.

In this study, 149 participants were diagnosed with T2DM and 168 participants were healthy without diabetes. Our cohort revealed a prevalence of 28% for *H. pylori* infection, which is lower than prior findings focusing on Middle Eastern countries. Previous studies have shown prevalence ranging from 45% to 65% in Middle Eastern countries including Egypt, Turkey, and Saudi Arabia, depending on population characteristics and diagnostic methods [26,38,39]. Our study, which employed different diagnostic methods and targeted population characteristics, may explain the lower prevalence at 28%. This demonstrates possible epidemiological variability in the region and highlights the requirement for assessments based on specific countries, especially for high-risk groups such as diabetic individuals.

In this research, a significant relationship was identified between diabetes status and *H. pylori* infection, with the chances of being *H. pylori*-positive 1.78 times higher in diabetic individuals. The findings are consistent with prior studies that revealed a potential connection between T2DM and *H. pylori* infection [13]. This relationship may reflect an underlying pathway involving changes in immune responses and gastrointestinal motility that are well-documented in diabetics [40]. From a biological standpoint, *H. pylori* may be significant in the pathogenesis of T2DM.

It is possible from a biological perspective that H. pylori plays a role in T2DM pathogenesis. There is an association between chronic *H. pylori* infection and systemic inflammation, as demonstrated by high levels of cytokines such as TNF-α, IL-6, and CRP [41]. The aforementioned inflammatory attributes have an adverse impact on insulin signaling through the promotion of serine phosphorylation of insulin receptor substrates (IRSs), which decreases insulin sensitivity and contributes to glucose dysregulation [42].

Additionally, it is possible for *H. pylori* infection to influence metabolic homeostasis via changes in the level of ghrelin and leptin, which regulate appetite and insulin responsiveness [43]. These proposed mechanisms are in accordance with findings by Mansori et al. (2020), whose meta-analysis demonstrated an association between *H. pylori* infection and higher diabetes risk (pooled OR ≈ 1.27 overall; ≈1.43 for Type 2 diabetes) [44].

Similar findings by Chen et al. (2019) [36] have shown increased HbA1c levels in patients diagnosed with *H. pylori* infection in comparison with patients who were not diagnosed with *H. pylori* (WMD = 0.50, 95% CI: 0.28–0.72, *p* < 0.001). Diabetic patients infected with *H. pylori* show higher HbA1c levels compared to those who are not infected with *H. pylori*, meaning that *H. pylori* infection may have an adverse impact on glycemic control [36]. Simultaneously, the data are aligned with a possible biological association between chronic *H. pylori* infection and insulin resistance.

It has been hypothesized that *H. pylori* colonization can initiate an inflammatory immune response, and chronic inflammation together with the insulin resistance it can induce may contribute to diabetes [45,46]. In the early stages of infection, neutrophils penetrate the gastrointestinal mucus [47], and once the infection has become chronic, monocytes replace them. The latter cells produce a variety of inflammatory cytokines, especially tumor necrosis factor-*α*, interleukin, and C-reactive protein, which have both local gastric and distal effects on other tissues/organs, contributing to several gastrointestinal conditions [48]. The insulin resistance resulting from these cytokines promotes diabetes, and their effects are compounded when the metabolic dysregulation of diabetes results in hyperglycemic, further contributing to the risk of long-term complications [49]. These inflammatory cytokines have been flagged in several studies as potential contributors to diabetes through *H. pylori* pathways [50].

There is a higher prevalence of diabetes in older adults, which is consistent with previous research demonstrating an increased risk of developing the disease with age as a consequence of insulin resistance and normal age-related changes in metabolism [51]. Diabetes was more prevalent in men than women and in participants with relatively high BMIs, both of which were in accordance with previous research, with males being at higher risk because of differences in fat distribution, lifestyle, and insulin sensitivity [52], while obesity has long been known to be a risk factor of the disease [53].

In terms of the trends of ABO blood group, national blood donor data in Jordan has shown that O blood group is the most common at around 38% to 44%, A blood group at 30% to 33%, B blood group at 15% to 18%, and AB blood group at 6% to 9%. In our sample, the ABO distribution largely reflects these known national blood group frequencies in Jordan, with group O being dominant. Thus, the higher number of individuals infected with O-type blood likely reflects the population’s demographic structure and not a true biological effect. Thus, the present research included an analysis of *H. pylori* infection prevalence in individuals with different ABO blood groups.

Our study included an analysis of the prevalence of *H. pylori* infection among participants with different ABO blood groups. This study found a small-to-moderate effect of blood group, with the highest prevalence of infection in participants with O-type blood (41.5%) and the lowest in those with A-type blood (19.1%). These findings are consistent with suggestions arising from earlier epidemiological studies [54,55] for an increased susceptibility to *H. pylori* colonization and infection in individuals with O-type blood. These findings are also consistent with research suggesting that individuals with O-type blood have a higher susceptibility to *H. pylori* infection and increased gastric cancer risk in A/AB groups [23].

A possible reason for the increased risk of *H. pylori* infection amongst those with blood group O concerns is the expression of certain Lewis blood group antigens and fucosylated oligosaccharides in the gastric mucosa, where *H. pylori* is able to bind to [56]. The BabA protein of *H. pylori* binds to one of the type 1 H antigens commonly found in the stomach lining [57] and are able to attach to the Le^b^ antigen, which is more abundant in individuals with O-type blood, thus facilitating colonization. The increased expression of the H antigen in individuals with O-type blood promotes bacterial binding and colonization of the stomach lining [58], which partially explains this blood group’s increased susceptibility to gastrointestinal diseases such as gastritis and stomach ulcers. The lower prevalence of infection associated with A-type blood may arise for many reasons, including differences in immune responses or mucosal antigenic profiles. There are undoubtedly many other factors that influence susceptibility to *H. pylori* infection (and some were examined in this study), including environment, hygiene practices, socioeconomic status, and genetics. Although these findings are fascinating, it is important to interpret them cautiously because this study employed cross-sectional comparisons. Jordan’s population structure may impact the distribution of H. pylori infection across ABO blood groups. In turn, more multilevel, population-adjusted studies are required in future to further elucidate the independent impact of ABO phenotype on infection risk.

The associations we examined between *H. pylori* infection and demographic/lifestyle factors highlighted the significantly increased susceptibility to infection among smokers, and this trend is in line with previous reports [59,60]. The risk of being infected with *H. pylori* was nearly twice as high for smokers compared to non-smokers, which may be due to smoking-induced alterations in the gastric mucosa that reduce its effectiveness as a barrier to microbial invasion and colonization [61,62]. Thus, it is particularly important to ensure that individuals’ smoking histories are properly assessed in public health initiatives aimed at controlling *H. pylori*.

There was a significantly higher *H. pylori* infection rate among individuals with lower levels of education. As noted earlier in this paper, education level correlates with many other factors such as poor living conditions and sanitation, lack of health knowledge, and low income. These factors are likely to be more directly related to the higher infection rates observed in this group—a point also made in several epidemiological studies that found similarly higher rates of infection in less educated and poorer social groups [63,64]. Health education must therefore be a priority in strategies aimed at reducing *H. pylori* infection rates. While our findings related to *H. pylori* infection and education (and the factors for which it serves as a proxy) are in accordance with previous studies, those relating to age, gender, and BMI were not. Earlier studies by Jonaitis and Nyssen [65] found infection rates to increase with time (and, therefore, age), but no such evidence was found in this study, which may be due to many reasons, including regional variations in *H. pylori* prevalence and the age distribution in our sample. Other research [66] has also observed associations between infection rates and BMI, attributing the association to metabolic and dietary differences among others, but this was not evident in our sample. Whether this was due to differences in the population sampled or simply reflects the lack of any consistent relationship remains unclear.

## 5. Conclusions

To conclude, this study found a statistically significant association between *H. pylori* infection and T2DM, in addition to an increased infection rate among O-blood type individuals within the study population. However, the cross-sectional nature of this study means that there is a correlation instead of causation in the interpretation of the findings. The present association highlights the need for further research considering biological, immunological, and socio-environmental influences. Possible future studies and mechanistic research are required in order to establish temporal direction and causality, especially pertaining to metabolic results and host genetic factors, including ABO blood grouping. The serological method adopted in this study detects IgG, which may take into account prior exposure instead of isolating the results to current infection. In addition, the assay’s moderate specificity of approximately 75.9% may result in certain degree of false-positive classification. It is therefore recommended that future studies adopt stool antigen and urea breath tests in order to improve detection of current infection and minimize any misclassification. Despite individuals with O-blood type having higher rates of *H. pylori* infection, the finding in this study reveals an association instead of establishing a causal link. Future possible or mechanistic studies are needed in order to establish if there is a direct association between O-blood type individuals and increased susceptibility to *H. pylori* infection.

Furthermore, there were several limitations in this study. Firstly, many confounding factors were not considered in the regression analysis, including total energy intake and level of physical activity, as these data were not recorded with the participant’s health details. In addition, a number of important clinical and socioeconomic variables were not included in the research, such as diabetes duration, hygiene conditions, socioeconomic status, and prior eradication treatment. In turn, this could limit the completeness of confounding adjustment. Future studies should thus include these variables in order to better understand the relationship between H. pylori infection and T2DM.

Secondly, we acknowledge that the sample size calculation relied on historical serology estimates instead of antigen-based prevalence, potentially compromising its accuracy. Nonetheless, the final sample size afforded sufficient statistical power for the observed effect sizes. To gain a better understanding of the relationships between the variables examined in this study and the biological mechanisms involved, future research should be conducted with larger, more diverse populations and to include more potentially significant factors using a multivariate approach.

Furthermore, false positives may occur with the application of rapid immunoassay, which has moderate specificity of 75.9%, potentially inflating the figures that suggest *H. pylori* infection is prevalent. In addition, prior exposure resulting in the persistence of IgG antibodies may prevent the assay from completely differentiating current and prior infection, adding to the possibility of misclassification bias. Exclusion cannot be made for the residual confounding from unmeasured variables, including comorbidities, nutritional status, and recent intake of antibiotics. Finally, it is not possible to totally remove recall bias. Patient-reported variables, including diabetes duration or previous antibiotic intake were obtained through self-reporting, where inaccurate recall may occur. Additionally, participant recruitment through tertiary hospital setting may lead to selection bias, since such population may be different from community-dwelling individuals in terms of disease severity, healthcare usage trends, and sociodemographic characteristics. Therefore, caution should be employed during the interpretation of these findings within the study, particularly those that pertains to Jordanians diagnosed with diabetes.

## Figures and Tables

**Table 1:** Participants’ demographic characteristic details.

	Diabetics (*n* = 149)	Non-Diabetics (*n* = 168)	Chi-Square Test	*p* Value
Gender				
Male	92 (61.7%)	87 (51.8%)	χ^2^ = 2.79	*p* = 0.09
Female	57 (38.3%)	81 (48.2%)		
Age Mean (SD)	57.8 (12.8)	43.1 (13.4)		*p* ≤ 0.05
20–29	3 (2.0%)	34 (20.2%)	χ^2^ = 78.73	*p* ≤ 0.05
30–39	10 (6.7%)	26 (15.4%)		
40–49	22 (15.4%)	56 (33.3%)		
50–59	39 (26.2%)	28 (16.7%)		
60–69	52 (34.9%)	22 (13.1%)		
70–79	16 (10.7%)	2 (1.2%)		
80+	6 (4.0%)	0 (0.0%)		
BMI Mean (SD)	30.77 (5.85)	28.45 (5.36)	t = 3.19	*p* ≤ 0.05
<18.5	2 (1.3%)	4 (2.4%)	χ^2^ = 7.61	*p* ≤ 0.05
18.5–24.9	22 (14.7%)	36 (21.4%)		
25–29.9	56 (37.6%)	72 (42.9%)		
30–34.9	43 (28.8%)	39 (23.2%)		
35–39.9	23 (15.4%)	16 (9.5%)		
40+	3 (2.0%)	1 (2.4%)		
Tobacco use				
Smoker	70 (47.0%)	60 (35.7%)	χ^2^ = 4.19	*p* ≤ 0.05
Non-Smoker	79 (53.0%)	108 (64.3%)		
Education level				
Illiterate	11 (7.4%)	2 (1.2%)	(χ^2^) = 42.92	*p* ≤ 0.05
Primary	30 (20.1%)	16 (9.5%)		
Secondary	49 (32.9%)	83 (49.4%)		
Bachelor’s	46 (30.9%)	66 (39.3%)		
Postgraduate	13 (8.7%)	1 (0.6%)		

**Table 2:** Odds ratios (ORs) with 95% confidence intervals (CIs) for each variable’s association with diabetes.

	Diabetics	Non-Diabetics	OR	*p* Value
Gender				
Male	92	87	1.5 (95% CI: 0.60–1.72)	*p* ≤ 0.05
Female	57	81	0.66	
Age				
20–29	3	34	0.096 (95% CI: 0.03–0.32)	*p* ≤ 0.05
30–39	10	26	0.38 (95% CI: 0.19–0.79)	*p* = 0.02
40–49	23	56	0.45 (95% CI: 0.27–0.74)	*p* ≤ 0.05
50–59	39	28	0.73 95% CI: 0.41–1.29)	*p* = 0.14
60–69	52	22	2.80 (95% CI: 1.69–4.66)	*p* ≤ 0.05
70–79	16	2	9.25 (95% CI: 2.12–40.35)	*p* ≤ 0.05
80+	6	0	14.1 (95% CI: 2.78–54.5)	*p* ≤ 0.05
BMI				
<18.5	2	4	0.56 (95% CI: 0.10–3.09)	*p* = 0.69
18.5–24.9	22	36	0.64 (95% CI: 0.35–1.14)	*p* = 0.15
25–29.9	56	72	0.80 (95% CI: 0.51–1.26)	*p* = 0.36
30–34.9	43	39	1.34 (95% CI: 0.81–2.22)	*p* = 0.30
35–39.9	23	16	1.73 (95% CI: 0.88–3.42)	*p* = 0.12
40+	3	1	3.43 (95% CI: 0.35–33.35)	*p* = 0.35
Tabaco use				
Smoker	70	60	1.60 (95% CI: 1.02–2.50)	*p* ≤ 0.05
Non-Smoker	79	108	0.62 (95% CI: 0.40–0.98)	*p* ≤ 0.05
Education level				
Illiterate	11	2	6.62 (95% CI: 1.44–30.35)	*p* ≤ 0.05
Primary	30	16	2.39 (95% CI: 1.25–4.60)	*p* ≤ 0.05
Secondary	49	83	0.50 (95% CI: 0.32–0.97)	*p* ≤ 0.05
Bachelor’s	46	66	0.69 (95% CI: 0.43–1.10)	*p* = 0.13
Postgraduate	13	1	15.96 (95% CI: 2.06–123.57)	*p* ≤ 0.05

**Table 3:** Logistic regression analyses of the factors associated with T2DM.

	Diabetics (*n* = 149)	Non-Diabetics (*n* = 168)	Logistic Regression *p* Value	
			OR (95%CI)	*p* Value
Gender				
Male	92	87	1.22 (95% CI: 0.89–1.66)	*p* = 0.44
Female	57	81		
Age				
20–29			0.11 (0.03–0.36)	*p* ≤ 0.05
30–39			0.29 (0.13–0.60)	*p* ≤ 0.05
40–49			0.39 (0.23–0.66)	*p* ≤ 0.05
50–59			0.71 (0.44–1.12)	*p* = 0.14
≥60 (reference)				
BMI				
<25 kg/m^2^ (reference)				
25–29.9 kg/m^2^			1.32 (0.86–2.02	*p* = 0.2
30–34.9 kg/m^2^			1.89 (1.12–3.18)	*p* ≤ 0.05
≥35 kg/m^2^			2.42 (1.21–4.86)	*p* ≤ 0.05
Tabaco use				
Smoker	70	60	1.51 (95% CI: 1.03–2.23)	*p* ≤ 0.05
Non-Smoker	79	108		
Education level				
School or less	90	101	2.28 (95% CI: 1.28–4.02)	*p* ≤ 0.05
University	59	67		

**Table 4:** Odds ratios (ORs) with 95% confidence intervals (CIs) for demographic and lifestyle variables associated with T2DM.

Variable	Diabetics (*n*)	Non-Diabetics (*n*)	OR (95% CI)/*p* Value
Age ≥ 60 y	68	24	3.12 (1.85–5.27); *p* ≤ 0.05
Smoker	70	60	1.60 (1.02–2.50); *p* ≤ 0.05
Education ≤ Secondary	90	101	1.56 (1.28–2.44); *p* ≤ 0.05
BMI ≥ 30 kg/m^2^	69	56	1.34 (0.81–2.22); *p* = 0.30

**Table 5:** Associations between T2DM and *H. pylori* infection status.

	Type 2 Diabetics (*n* = 149)	Non-Diabetics (*n* = 168)	Chi-Square	df	*p* Value	OR	Cramer’s V
Positive for *H. pylori* (*n* = 89)	51 (34.2%)	38 (22.6%)	4.711	1	≤0.05	1.78 (1.08–2.92)	*p* = 0.122
Negative for *H. pylori* (*n* = 228)	98 (65.7%)	130 (77.6%)					

**Table 6:** Association between *H. pylori* status and ABO blood group.

Subcategory	*H. pylori*_Pos (*n* = 89)	*H. pylori*_Neg (*n* = 228)	Chi-Square	df	*p* Value	Cramer’s Value
A	21 (23.6%)	89 (37.7%)	15.01	3	<0.001	0.218, small-to-moderate association
B	13 (14.6%)	46 (18.4%)				
AB	11 (12.6%)	31 (12.3%)				
O	44 (49.4%)	62 (26.7%)				

## Data Availability

The datasets used and/or analyzed during the current study available from the corresponding author on reasonable request.

## Supplementary Materials

The following supporting information can be downloaded at: https://www.mdpi.com/article/10.3390/medicina61122167/s1, Table S1: Demographic queassionaier used in this study; Supplementary Figure S1: Representative results of the DiaSpot^®^ *H. pylori* One-Step Test (PT Indo DiaSpot, Indonesia). Each cartridge includes a control (C) and test (T) line; test sensitivity 95.9%, specificity 75.9%.
