# Supplementary material for: The Prevalence of H. pylori Among Jordanian Type 2 Diabetic Patients and Its Association with ABO Blood Group
Source: Medicina (Kaunas). 2025 Dec 5;61(12):2167. doi: 10.3390/medicina61122167 (PMC12734858; doi:10.3390/medicina61122167)
Supplement: Supplementary file 1 [file medicina-61-02167-s001.zip › medicina-3923324-supplementary.pdf]

Article

The Prevalence of *H. pylori* Among Jordanian Type 2 Diabetic Patients and Its Association with ABO Blood Group

Hafez Al-Momani [1,*], Amro Bani-Hani [2], Ahmad A. Jaber [2], Azhar Alsmady [2], Yusra Sobh [2], Bassam Otoom [2], Iman Aolymat [3], Ashraf I. Khasawneh [1], Hala Tabl [1], Ayman Alsheikh [4], AbdelRahman M. Zueter [5] and Abdel-Ellah Al-Shudifat [6]

[1] Department of Microbiology, Pathology and Forensic Medicine, Faculty of Medicine, The Hashemite University, Zarqa 13133, Jordan; ashrafkh@hu.edu.jo (A.I.K.); halaa_mo@hu.edu.jo (H.T.)

[2] Faculty of Medicine, The Hashemite University, Zarqa 13133, Jordan; dr.amrofbh@gmail.com (A.B.-H.); amdjbr99@gmail.com (A.A.J.); azharalsmady@gmail.com (A.A.); sobuhu@gmail.com (Y.S.); bassams.otoom123@gmail.com (B.O.)

[3] Department of Anatomy, Physiology and Biochemistry, Faculty of Medicine, The Hashemite University, Zarqa 13133, Jordan; imank@hu.edu.jo

[4] Department of Medical Laboratory Sciences, Faculty of Allied Medical Sciences, Zarqa University, Zarqa 13132, Jordan; asheikh@zu.edu.jo

[5] Department of Medical Laboratory Sciences, Faculty of Applied Medical Sciences, The Hashemite University, Zarqa 13133, Jordan; zeuterabdelrahman@gmail.com

[6] Department of Internal Medicine, Neurology, Psychiatry and Dermatology, Faculty of Medicine, The Hashemite University, Zarqa 13133, Jordan; abdel-ellah@hu.edu.jo

* **Correspondence: hafez@hu.edu.jo**

Gender	Male	Female
Age groups (Years)		
19-29		
30-39		
40-49		
50-59		
60-69		
70-79		

80 and over		
Weight (kg)		
Height (cm)		
Tobacco use	smoker	Non smokers
Educational level		
Illiterate		
Primary		
Secondary		
Bachelor's		
Postgraduate		

Supplementary table S1: Demographic queassionaier used in this study

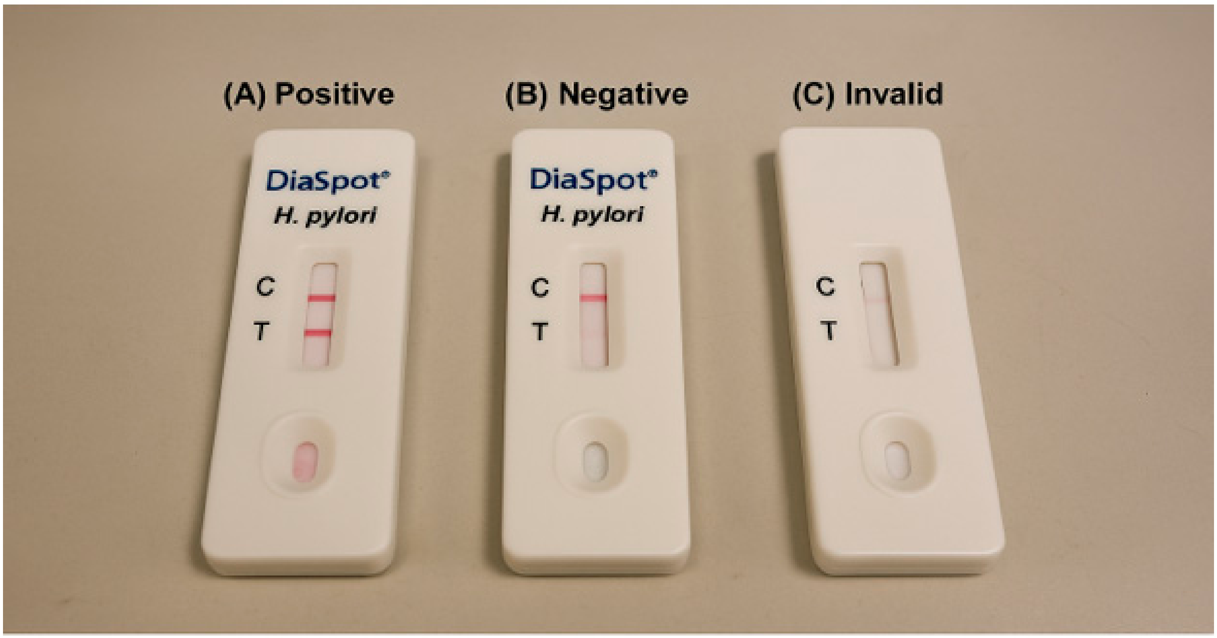

Supplementary Figure S1: Representative results of the DiaSpot® *H. pylori* One-Step Test (PT Indo DiaSpot, Indonesia). Each cartridge includes a control (C) and test (T) line; test sensitivity 95.9%, specificity 75.9%.”
